# Soluble proteins of chemical communication: an overview across arthropods

**DOI:** 10.3389/fphys.2014.00320

**Published:** 2014-08-27

**Authors:** Paolo Pelosi, Immacolata Iovinella, Antonio Felicioli, Francesca R. Dani

**Affiliations:** ^1^State Key Laboratory for Biology of Plant Diseases and Insect Pests, Institute of Plant Protection, Chinese Academy of Agricultural SciencesBeijing, China; ^2^Biology Department, University of FirenzeFirenze, Italy; ^3^Department of Veterinary Sciences, University of PisaPisa, Italy; ^4^CISM, Mass Spectrometry Centre, University of FirenzeFirenze, Italy

**Keywords:** odorant-binding proteins, chemosensory proteins, Niemann-Pick type C2 proteins, Insect olfaction, basal hexapods, arthropod chemoreception

## Abstract

Detection of chemical signals both in insects and in vertebrates is mediated by soluble proteins, highly concentrated in olfactory organs, which bind semiochemicals and activate, with still largely unknown mechanisms, specific chemoreceptors. The same proteins are often found in structures where pheromones are synthesized and released, where they likely perform a second role in solubilizing and delivering chemical messengers in the environment. A single class of soluble polypeptides, called Odorant-Binding Proteins (OBPs) is known in vertebrates, while two have been identified in insects, OBPs and CSPs (Chemosensory Proteins). Despite their common name, OBPs of vertebrates bear no structural similarity with those of insects. We observed that in arthropods OBPs are strictly limited to insects, while a few members of the CSP family have been found in crustacean and other arthropods, where however, based on their very limited numbers, a function in chemical communication seems unlikely. The question we address in this review is whether another class of soluble proteins may have been adopted by other arthropods to perform the role of OBPs and CSPs in insects. We propose that lipid-transporter proteins of the Niemann-Pick type C2 family could represent likely candidates and report the results of an analysis of their sequences in representative species of different arthropods.

## Soluble binding proteins in detection and delivery of semiochemicals

Odor detection is accomplished in vertebrates as in insects through a complex and sophisticated sensory system making use of both membrane-bound receptors (Buck and Axel, 1991; Clyne et al., 1999; Vosshall et al., 1999) and soluble binding proteins (Pelosi et al., 1981, 1982; Vogt and Riddiford, 1981). These latter are commonly regarded as solubilizers and carriers of odorants and pheromones, generally hydrophobic compounds, but in recent times evidence has been provided in some insect species for more specific and important roles. In particular, knock-out experiments have demonstrated that an OBP of *Drosophila melanogaster*, LUSH, is required for olfaction (Xu et al., 2005; Laughlin et al., 2008), while behavior assays with *Drosophila* mutants (Matsuo et al., 2007; Swarup et al., 2011) and with aphids (Qiao et al., 2009; Sun et al., 2012a) have indicated that OBPs are involved in semiochemical discrimination.

Odorant-binding proteins (OBPs) is the name designating two structurally unrelated families of polypeptides abundantly secreted into the nasal mucus of vertebrates and in the lymph of chemosensilla in insects. OBPs of vertebrates contain 150-160 amino acids (Bignetti et al., 1985; Pevsner et al., 1985; Pelosi, 1994, 1996; Tegoni et al., 2000) and belong to the superfamily of lipocalins (Flower, 1996, 2000), carrier proteins folded in the typical β-barrel shape, with eight β-sheets and one short segment of α-helix close to the C-terminus (Bianchet et al., 1996; Tegoni et al., 1996). OBPs of insects (around 130–140 residues), instead (Vogt and Riddiford, 1981; Pelosi et al., 2006; Vieira and Rozas, 2011; Leal, 2013), are made of six α-helical domains assembled in a compact and stable structure (Sandler et al., 2000; Tegoni et al., 2004). They are characterized by a pattern of six conserved cysteines paired into three interlocked disulfide bridges (Leal et al., 1999; Scaloni et al., 1999).

A third class of soluble binding proteins, named Chemosensory Proteins (CSPs) is also found in olfactory and gustatory organs of insects (McKenna et al., 1994; Pikielny et al., 1994; Angeli et al., 1999; Wanner et al., 2004; Pelosi et al., 2006; Vieira and Rozas, 2011). CSPs are around 100–120 residues long and present a conserved pattern of four cysteines forming two independent loops (Angeli et al., 1999). CSPs are also made of α-helical segments, but assembled in a folding different from that of insect OBPs (Lartigue et al., 2002; Tomaselli et al., 2006; Jansen et al., 2007).

**Figure 1:**
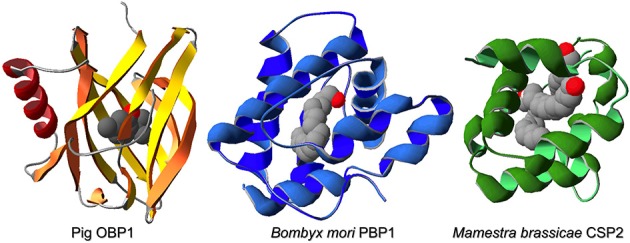
**Three-dimensional structures of pig OBP (PDB: 1E06, Vincent et al., 2000), *Bombyx mori* PBP1 (PDB: 1DQE, Sandler et al., 2000), and *Mamestra brassicae* CSP2 (PDB: 1N8U, Campanacci et al., 2003), representative examples of a vertebrate OBP, an insect OBP and an insect CSP, respectively**. Proteins of these three classes, despite marked structural differences, perform similar roles of transport and solubilization of semiochemicals and are extremely compact and stable. Structures have been visualized using the Swiss-Model PDB Viewer (Guex and Peitsch, 1997).

All three classes of soluble proteins contain hydrophobic binding pockets and, despite their structural differences, are believed to perform similar roles in vertebrates and in insects (Pelosi and Maida, 1990; Calvello et al., 2003). Figure 1 reports the three-dimensional folding of a representative protein from each of the three classes.

All three families of proteins, although generally associated with chemodetection, include members expressed outside chemosensory organs.

In vertebrates this phenomenon appeared clear soon after the discovery of the first OBP. In fact, it had already been known for long time that mice and rats excrete small proteins into their urine at concentrations of few milligrams per milliliter (Dinh et al., 1965; Finlayson et al., 1965), but a reasonable explanation for this large waste of energy was not proposed until sequence information was obtained for the bovine OBP, the first to be discovered, which showed high similarity with urinary proteins (Cavaggioni et al., 1987; Cavaggioni and Mucignat-Caretta, 2000). Some proteins were found both in the nasal mucosa of the mouse and in the urine, with the only difference that when secreted into urine they were loaded with species-specific pheromones (Bacchini et al., 1992; Robertson et al., 1993). More examples of mammalian OBPs involved in semiochemical delivery are the boar salivary lipocalins (Marchese et al., 1998; Loebel et al., 2000; Spinelli et al., 2002), the hamster aphrodisin (Singer et al., 1986; Vincent et al., 2001) and the horse sweat lipocalin Equ-c1 (D'Innocenzo et al., 2006). In addition, a lipocalin related to OBPs, the apolipoprotein D, was reported in human sweat, complexed with a volatile fatty acid (Zeng et al., 1996). In all these cases OBPs have been found complexed with pheromones, strongly supporting a function in semiochemical delivery. While it is reasonable to assume that the same or similar proteins might be involved in the dual role of detecting and releasing chemical signals, functions unrelated to chemical communication would appear less obvious. However, if we consider the phenomenon under a wider perspective, we realize that the superfamily of lipocalins, to which vertebrate OBPs belong, includes many different members endowed with diverse functions. In fact, all lipocalins, despite major differences in amino acid sequences, share a conserved architecture (Flower, 1996; Flower et al., 2000). The reason for such versatility of lipocalins is to be found in their extremely stable and compact structure, which allowed adaptation to various uses in different and often challenging conditions.

In insects, a similar phenomenon has only been described in recent years, but several pieces of evidence have been rapidly accumulating in different species. Both OBPs and CSPs have been detected in pheromone glands or in reproductive organs, where they might assist releasing of semiochemicals into the environment. Typical examples are the CSPs found in the pheromone glands of *Mamestra brassicae* (Jacquin-Joly et al., 2001), *Bombyx mori* (Dani et al., 2011) and *Agrotis ipsilon* (Gu et al., 2013), as well as the OBP10 of *Helicoverpa armigera* and *H. assulta* (Sun et al., 2012b), the OBP22 of *Aedes aegypti* (Li et al., 2008) and the CSP91 of *Locusta migratoria*, produced in the male seminal fluid and transferred to the female, likely with a bound pheromone, during mating (Ban et al., 2013; Zhou et al., 2013). Several OBPs and CSPs are also expressed in the mandibular glands of the honey bee (Iovinella et al., 2011). Other members of both classes seem to be involved in roles completely different from chemical communication. It is noteworthy that the first member of the CSP family, named p10, was discovered in a context not related to chemical communication, as a protein involved in limb regeneration in the cockroach (Nomura et al., 1992; Kitabayashi et al., 1998). Other representative examples of CSPs performing roles unrelated to chemical communication are the CSP5 of the honeybee, only found in ovaries and eggs and required for development of the embryo (Maleszka et al., 2007), and the CSP4 of *Helicoverpa armigera* and *H. assulta*, present in high concentration in the lumen of the proboscis and likely acting as a surfactant to help sucking (Liu et al., 2014a). Recently, CSPs have been also indicated as possible scavengers for insecticides, thus providing insects with a sort of resistance to their lethal effects (Liu et al., 2014b; Xuan et al., 2014). Some OBPs have also been related to roles other than chemoreception: OBP56a is expressed in the oral disk of the house fly *Phormia regina* and has been reported as a fatty acid solubilizer (Ishida et al., 2013). Other examples are two “tandem OBPs” (resulting from two different OBPs joined by a bridge of few amino acids) of the mosquito *Aedes aegypti*. The first is the salivary protein D7r4, which is involved in antiinflammatory processes (Calvo et al., 2009), the second is the OBP45, reported in the ovaries and eggs with a putative function in reproductive mechanisms responsible for oocyte maturation (Costa-da-Silva et al., 2013; Marinotti et al., 2014).

**Figure 2:**
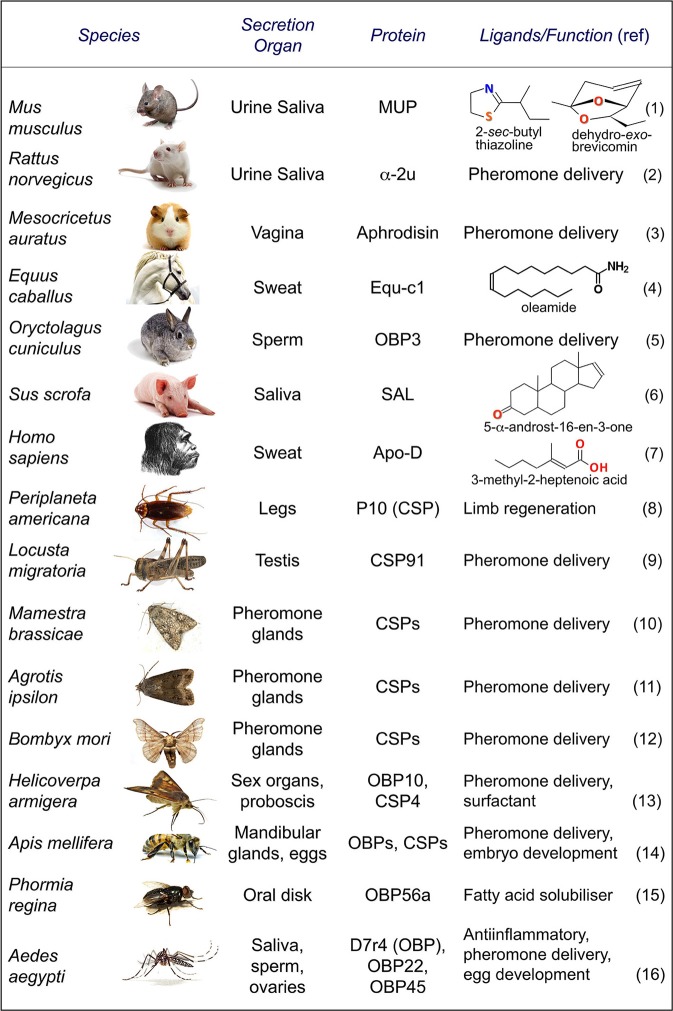
**OBPs and CSPs involved in non-sensory functions**. Mammalian OBPs have been found in secretions involved in the delivery of semiochemicals. In several cases, when isolated from such biological fluids, OBPs carry species-specific pheromones. Insect OBPs and CSPs have been reported both in pheromone glands and in reproductive organs, where they likely solubilize and bind specific pheromones. Moreover, members of both classes have been reported in other tissues and shown to be involved in functions unrelated to chemical communication. (1) Finlayson et al., 1965; Bacchini et al., 1992; (2) Dinh et al., 1965; (3) Singer et al., 1986; (4) D'Innocenzo et al., 2006; (5) Mastrogiacomo et al., 2014; (6) Marchese et al., 1998; (7) Zeng et al., 1996; (8) Nomura et al., 1992; Kitabayashi et al., 1998; (9) Zhou et al., 2013; (10) Jacquin-Joly et al., 2001; (11) Gu et al., 2013; (12) Dani et al., 2011; (13) Sun et al., 2012b; Liu et al., 2014a; (14) Iovinella et al., 2011; Maleszka et al., 2007; (15) Ishida et al., 2013; (16) Calvo et al., 2009; Costa-da-Silva et al., 2013; Marinotti et al., 2014; Li et al., 2008.

Figure 2 lists representative cases of OBPs (both in vertebrates and in insects) and CSPs utilized for tasks other than chemodetection.

As observed with lipocalins, compact folding and stability are the characteristics on the basis of such diverse uses of insects OBPs and CSPs, resulting in extreme refractivity to heat, chemicals and proteolytic enzymes (Paolini et al., 1999; Ban et al., 2002; Schwaighofer et al., 2014). Besides, they all present hydrophobic pockets for small and medium size organic chemicals. Therefore, it is reasonable to think that such stable and efficient binding proteins have been utilized for different tasks in various organs, wherever there was need to transport hydrophobic chemicals in aqueous media or to protect some compounds from degradation or else to assure a gradual release of semiochemicals in the environment.

Although such high versatility is associated with all three classes of binding proteins, we can suggest that the structure of insect OBPs is probably the least adaptable to perform different functions, based on the low number of insect OBPs so far reported to perform non-chemosensory functions, with respect to CSPs and vertebrate OBPs. In fact, the folding of insect OBPs is strongly constrained by its three interlocked disulfide bridges, as opposed to more flexible CSPs, which possess two separate bridges, and to vertebrate OBPs, whose β-barrel can swell to a relatively large extent, thus offering diverse structural solutions within a similar folding motif.

An important consequence of this phenomenon for discussing the data that will be presented further on is that sequence similarity alone or the assignment of a new member to one of the three families of binding proteins does not necessarily imply an involvement in chemosensing or in chemical communication.

This review focuses on soluble olfactory proteins of insects with a broader view across all arthropods, in the attempt to outline their possible evolution.

## OBPs and CSPs across evolution of insects and arthropods

Given the structural differences between vertebrate OBPs, insects OBPs and CSPs, it is clear that these three families of proteins followed independent evolutionary paths. In particular, it would be of interest to trace down the onset of insect OBPs and CSPs and possibly identify their likely progenitor genes.

So far, insect OBPs have only been described in insects, while some CSPs have been reported in other arthropods (Forêt et al., 2007; Iovinella et al., 2013), although their involvement in chemical communication in non-insect species has not been demonstrated.

Therefore, we have searched for members of these two classes of proteins in basal hexapods (Giribet et al., 2004) and in other arthropods. For those species whose genome has been sequenced we are in the condition of stating whether or not OBPs and CSPs exist and to report the number of their genes. For other species, our search was performed by blasting the EST database using as queries the sequences of species phylogenetically (Giribet and Edgecombe, 2012) as close as possible to those under investigation.

### Odorant-binding proteins in insects and arthropods

OBPs have been reported in a large number and variety of insect species (Vieira and Rozas, 2011). The number of their genes in species that had their genome sequenced ranges from a dozen in some ant species (Smith et al., 2011) to more than hundred in some mosquitoes (Manoharan et al., 2013). OBPs are extremely divergent in their sequences and identical amino acids between members of the same species, as well as between species, may be even lower than 10%. The correct assignment of a sequence to the family of OBPs is mainly based on the conserved pattern of six cysteines, determining with their three interlocked bridges the folding and the stability of these proteins. However, OBPs with four cysteines (C-minus OBPs) or with a larger number of such residues (C-plus OBPs) have been reported in many species (Zhou et al., 2004). Based on such criteria and data, the assignment of a new protein to the class of OBPs can be performed with reasonable confidence.

Therefore, using BLAST search tools, we have looked for the presence of OBPs in basal hexapods and in other arthropods. In particular, we have searched protein databases, nucleotide collections and EST sequences in the following groups: Collembola, Diplura, Protura, Archaeognatha, and Zygentoma.

Our search only produced a total of 7 sequences in two species of Collembola, and a single sequence in a Zygentoma species, while we could not detect any gene similar to OBPs in the other groups.

A similar search could not yield any sequence recognizable as OBP in other arthropods, namely Crustacea, Myriapoda, and Chelicerata, as well as in the sister groups Onychophora and Tardigrada. In particular, we used as templates the OBPs of *Locusta migratoria*, *Acyrthosiphon pisum*, *Bombyx mori*, as well as those found in basal hexapods as part of this work. As for some of the species under analysis, such as *Daphnia pulex*, *Ixodes scapularis*, and *Varroa destructor*, partial or complete genome information is available, we can reasonably assume that the class of proteins defined as “insect OBPs” is only found in hexapods. The few OBP genes detected in basal hexapods, such as Collembola and Zygentoma, indicate that OBPs are present since the very first differentiation of the Hexapoda. How these more efficient proteins originated is still unknown, as we were not able to find sequences that might appear as progenitors in other species of arthropods.

**Figure 3:**
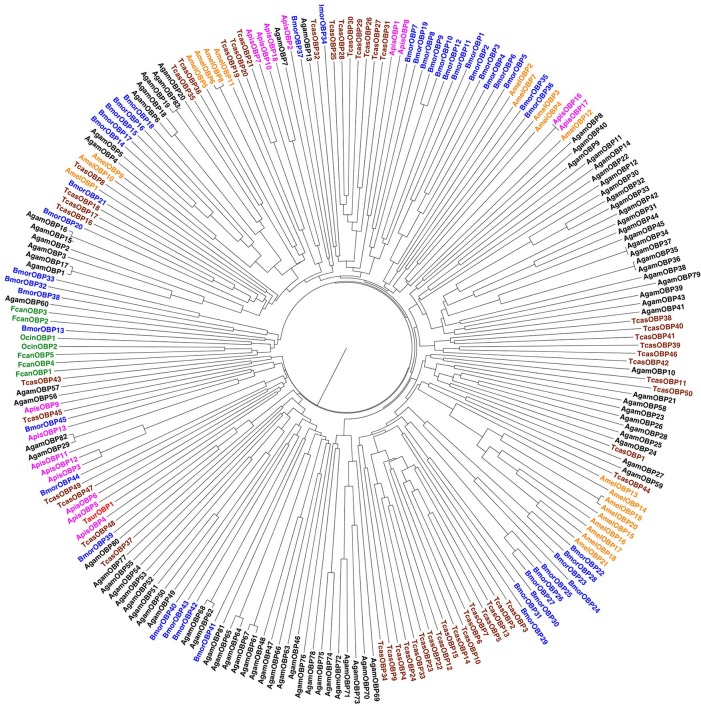
**Phylogenetic tree of OBPs from selected species of insects and basal hexapods**. Among arthropods, OBPs were only found in Hexapoda. Species and color codes are as follows. Red: Zygentoma (Taur: *Thricolepisma aurea*); green: Collembola (Fcan: *Folsomia candida*; Ocin: *Orchesella cincta*); magenta: Hemiptera (Apis: *Acyrthosiphon pisum*); brown: Coleoptera (Tcas: *Tribolium castaneum*); blue: Lepidoptera (Bmor: *Bombyx mori*); orange: Hymenoptera (Amel: *Apis mellifera*); black: Diptera (Agam: *Anopheles gambiae*). Sequences were aligned with the on-line software Clustal-W, using the following parameters. For Pairwise alignment: Protein Weight Matrix: Gonnet; Gap open: 10; Gap extension: 0.1. For Multiple sequence alignment: Protein Weight Matrix: Gonnet; Gap open: 10; Gap extension: 0.2; Gap distance: 5; Clustering: NJ. Phylogenetic trees were visualized with the software Fig Tree (http://tree.bio.ed.ac.uk/software/figtree/). Accession numbers are taken from Vieira and Rozas, 2011.

Figure 3 reports a phylogenetic tree built with all the OBPs of selected insect species among those whose genome is available, together with the few members found in Collembola and Zygentoma. More information on the number of OBPs in each species can be found in Table 1. Although the sample of sequences relative to basal hexapods is too small to allow any conclusion, nevertheless we can observe that the five sequences of the collembolan *Folsomia candida* exhibit a wide divergence, as is the case with other insects OBPs, indicating that their differences can possibly cope with the diversity of semiochemicals in the environment.

**Table 1:** **List of species examined in this work with the number of OBPs, CSPs, and NPC2s so far detected in the databases**.

**Taxon**	**Order**	**Species**	**OBP**	**CSP**	**NPC2**
**TARDIGRADA**					
		*Hypsibius dujardini*	–	–	4
**ONYCHOPHORA**					
		*Peripatopsis sedgwicki*	–	–	1
		*Epiperipatus* sp.	–	–	1
**EUCHELICERATA**					
Arachnida	Xiphosura	*Limulus polyphemus*	–	–	2
	Tetrapulmonata, Araneae	*Latrodectus hesperus*	–	–	1
		*Loxosceles laeta*	–	–	1
	Scorpiones	*Hottentotta judaicus*	–	–	1
	Acari	*Ixodes scapularis*	–	1	14
**MYRIAPODA**					
	Diplopoda	*Julida* sp.	–	2	–
		*Archispirostreptus gigas*	–	2	–
**CRUSTACEA**					
	Branchiopoda	*Artemia franciscana*	–	1	4
		*Daphnia pulex*		1	12
		*Triops cancriformis*	–	2	3
**HEXAPODA**					
Entognatha	Diplura	*Campodea fragilis*	–	–	3
	Protura	*Acerentomon franzi*	–	–	2
	Collembola	*Folsomia candida*	4	1	4
		*Anurida maritima*	–	1	6
		*Onychiurus arcticus*	–	–	11
		*Cryptopygus antarcticus*	–	2	–
		*Orchesella cincta*	2	–	–
Ectognatha (Insecta)	Archeognatha	*Lepismachilis y-signata*	–	2	–
	Zygentoma	*Thricolepisma aurea*	1	1	–
	Orthoptera	*Locusta migratoria*	22	70	2
	Hemiptera	*Acyrthosiphon pisum*	16	12	2
	Coleoptera	*Tribolium castaneum*	50	19	9
	Lepidoptera	*Bombyx mori*	45	16	8
	Hymenoptera	*Apis mellifera*	21	6	5
		*Megachile rotundata*	7	7	4
		*Nasonia vitripennis*	90	10	5
	Diptera	*Drosophila melanogaster*	52	4	7
		*Culex quinquefasciatus*	109	27	13
		*Anopheles gambiae*	69	8	6

*In species where the genome has been published, these figures can be considered as more or less final, in other species their numbers could increase as more information will become available. We have adopted the classification reported in **Figure 5** of Giribet and Edgecombe (2012), who suggest that Mandibulata include Myriapoda, Crustacea, and Hexapoda*.

### Chemosensory proteins in insects and arthropods

Chemosensory proteins are better conserved than OBPs across insect species and can be found in several other arthropods, including Crustacea, Myriapoda, and Euchelicerata. With respect to OBPs, CSPs are in general more widely expressed in different parts of the body, suggestive often of nonspecific functions. As already observed for OBPs, also the number of CSP genes in different species of insects is highly variable, from as few as four in *D. melanogaster* (Vieira and Rozas, 2011) to at least 70 in *L. migratoria* (Zhou et al., 2013). Although proteins of both classes, owing to their successful folding and stability, are utilized for different tasks besides chemical communication, it is true that most of the studies on OBPs have been associated with chemoreception, while often CSPs have been reported in connection with other physiological events. The exceptional versatility of CSPs might be related to their high capacity of accepting ligands of different sizes. At least in one case, X-ray crystallography has demonstrated that a CSP can swell to a large extent and bind three molecules of 12-Br-dodecanol (Campanacci et al., 2003). The same adaptability is probably the structural reason why CSPs have been adopted in more than one species as scavengers for insecticides of largely different sizes, such as avermectin (Xuan et al., 2014) and thiametoxam (Liu et al., 2014b).

**Figure 4:**
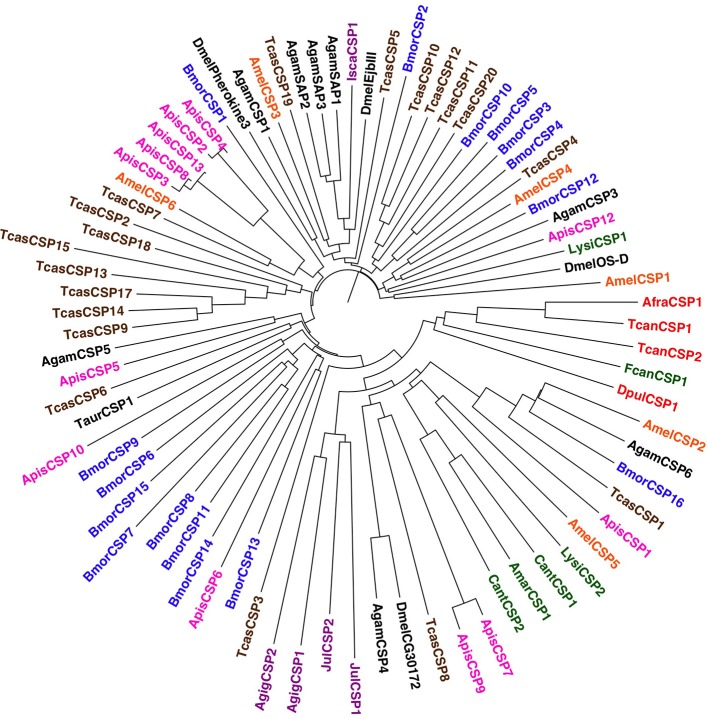
**Phylogenetic tree of CSPs from selected species of insects and other arthropods**. Apart from Hexapoda, members of the CSP family have also been found in species of Euchelicerata, Myriapoda, and Crustacea. Species and color codes are as follows. Violet: Euchelicerata (Isca: *Ixodes scapularis*) and Myriapoda (Jul: *Julida* sp.; Agig: *Archispirostrepsus gigas*); red: Crustacea (Afra: *Artemia franciscana*; Dpul: *Daphnia pulex*; Tcan: *Triops cancriformis*); green: Collembola (Fcan: *Folsomia candida*; Amar: *Anurida maritima*; Cant: *Cryptopygus antarcticus*; Ocin: *Orchesella cincta*), Archaeognatha (Lysi: *Lepismachilis y-signata*) and Zygentoma (Taur: *Thricolepisma aurea*); magenta: Hemiptera (Apis: *Acyrthosiphon pisum*); brown: Coleoptera (Tcas: *Tribolium castaneum*); blue: Lepidoptera (Bmor: *Bombyx mori*); orange: Hymenoptera (Amel: *Apis mellifera*); black: Diptera (Dmel: *Drosophila melanogaster*, Agam: *Anopheles gambiae*). Sequences were aligned and trees were visualized as in Figure 2. Accession numbers are taken from Vieira and Rozas (2011) or are reported in Table S1.

Given such wide repertoire of functions in which CSPs can be involved, it is more difficult to track the use of these proteins in chemodetection across arthropods. To provide a comprehensive picture of CSPs, a phylogenetic tree built on the sequences from selected insect species and all those found so far in other arthropods is reported in Figure 4. More detailed data are provided in Table 1.

As a matter of fact, genes encoding CSPs have been found in arthropods other than insects (Pelosi et al., 2006; Zhou et al., 2006), but it would be hard to state that these proteins take the place of OBPs in those species. In fact, no more than one or two sequences have been detected in each species, even when, as in the case of *Daphnia pulex*, full genomic information is available. It would be more reasonable to link the presence of these CSPs to other functions, such as development. This view is also suggested by the fact that all CSPs of basal hexapods and non-insect arthropods, with the only exception of the single sequence of *I. scapularis*, cluster in the same large group, together with CSP5 of the honeybee, a protein only found in ovaries and eggs and shown, using experiments of RNA interference, to be required for a correct development of the embryo (Maleszka et al., 2007). Another few insect CSPs, of so far unknown functions, are found in the same branch of the tree; it would be tempting to speculate that perhaps they could also be involved in development or other roles. On the other hand, the single CSP of *I. scapularis*, so far identified, clusters with three members of the mosquito *A. gambiae* (SAP1-SAP3) specifically expressed in antennae (Mastrobuoni et al., 2013) and reported to bind several odorants (Iovinella et al., 2013).

Taken together, the information available so far suggests that a role of CSPs in chemodetection, similar to that reported for OBPs, can only be recognized in insects.

Therefore, while insects make use of both OBPs and CSPs in chemical communication, we are left with no candidates for analogous roles in other arthropods.

## A new putative class of transport proteins for semiochemicals

To identify suitable candidate proteins in other arthropods which might perform the roles of OBPs and CSPS in Hexapoda chemical communication, we searched among the available databases for other families of binding proteins using the following criteria and guidelines:

- there should be a sufficient number of genes in the same species (probably at least a dozen, taking as a reference the 12 OBPs of some ant species), to ensure recognition of complex chemical stimuli using a “combinatorial code” (Malnic et al., 1999);
- similarly to OBPs and CSPs, these proteins should be small and soluble;
- their structure should include a hydrophobic binding pocket;
- being in contact with the external environment, they should be extremely stable to temperature, chemical agents and proteolysis, as much as OBPs and CSPs are.

This last characteristic brings an important consequence which greatly helped our search: small stable proteins are often powerful allergens, as they can reach the blood stream unchanged or slightly affected and trigger immune responses. It is well-known that most lipocalins are allergens, the best examples being β-lactoglobulin (Mäntyjärvi et al., 2000) and several mammalian OBPs, such as Equ-c1, highly abundant in horse sweat (D'Innocenzo et al., 2006). In fact, quite a number of proteins, first reported as allergens, turned out to be members of the mammalian OBP family.

Based on these considerations, we searched for families of allergens presenting a small size and compact structure. We first analyzed the genome of the tick *I. scapularis* and found 14 sequences classified as Nieman-Pick proteins, type C2 (NPC2), which could fulfill our criteria.

### NPC2 in vertebrates

**Figure 5:**
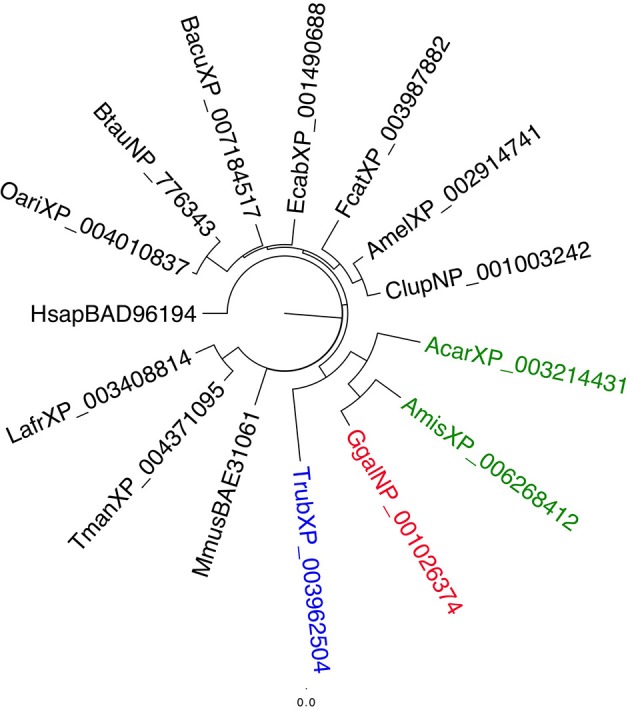
**Phylogenetic tree of NPC2 proteins from selected species of vertebrates**. These proteins are highly conserved in vertebrates and only a single gene is present in each species. Their role is to bind and transport cholesterol and other lipids. Species and color codes are as follows. Green: Reptiles (Acar: *Anolis carolinensis*; Amis: *Alligator mississippiensis*); blue: Fishes (Trub: *Takifugu rubripes*); red: Birds (Ggal: *Gallus gallus*); black: Mammals (Mmus: *Mus musculus*; Fcat: *Felis catus*; Clup: *Canis lupus*; Btau: *Bos taurus*; Ecab: *Equus caballus*; Oari: *Ovis aries*; Amel: *Ailuropoda melanoleuca*; Lafr: *Loxodonta africana*; Bacu: *Balaenoptera acutorostrata*; Tman: *Trichechus manatus*; Hsap: *Homo sapiens*). Sequences were aligned and trees were visualized as in Figure 2. Names of sequences include accession numbers.

This family of proteins has been widely studied in vertebrates, where they are associated with cholesterol and lipid binding and trafficking (Storch and Xu, 2009). A search through the databases has returned only a single sequence per species of mammals and other vertebrates. Figure 5 reports a phylogenetic tree of NPC2 from representative vertebrates. Another characteristic of NPC2 of vertebrates is their high conservation across species, with identity values higher than 75% between mammalians and around 55–70% between mammals and other vertebrates. This is hardly surprising if their function is the same in all species, mainly to bind cholesterol and lipids.

### NPC2 in arthropods

During the course of our search, a paper reporting the expression of a member of the NPC2 family in the antennae of the ant *Camponotus japonicus* (Ishida et al., 2014), supported our hypothesis that such proteins could be involved in chemodetection.

**Figure 6:**
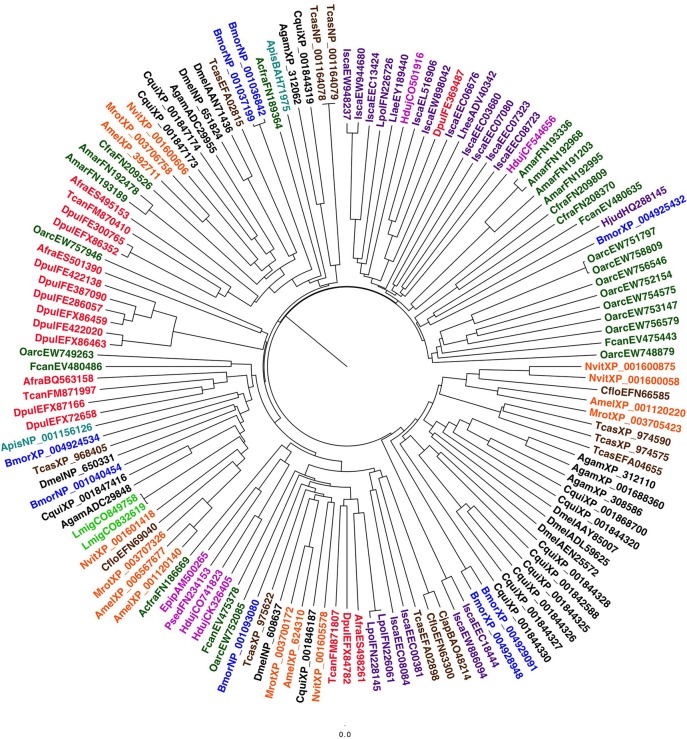
**Similarity tree of Npc2s from selected species of insects and other arthropods**. Phylogenetic tree of NPC2 proteins from selected species of insects and other arthropods, as well as “sister groups.” Species and color codes are as follows: magenta: Tardigrada (Hduj: *Hypsibius dujardini*) and Onychophora (Psed: *Peripatopsis sedgwicki*; Epip: *Epiperipatus* sp.); violet: Euchelicerata (Lpol: *Limulus polyphemus*; Lhesp: *Latrodectus hesperus*; Llae: *Loxosceles laeta*; Hjud: *Hottentotta judaicus*; Isca: *Ixodes scapularis*); red: Crustacea (Afra: *Artemia franciscana*; Dpul: *Daphnia pulex*; Tcan: *Triops cancriformis*); green: Collembola (Acfra: *Acerentomon franzi*; Fcan: *Folsomia candida*; Amar: *Anurida maritima*; Oarc: *Onychiurus arcticus*); light green: Orthoptera (Lmig: *Locusta migratoria*); light blue: Hemiptera (Apis: *Acyrthosiphon pisum*); brown: Coleoptera (Tcas: *Tribolium castaneum*; Cjap: *Camponotus japonicus*; Cflo: *Camponotus floridanus*); blue: Lepidoptera (Bmor: *Bombyx mori*); orange: Hymenoptera (Amel: *Apis mellifera*; Mrot: *Megachile rotundata*; Nvit: *Nasonia vitripennis*); black: Diptera (Dmel: *Drosophila melanogaster*, Cqui: *Culex quinquefasciatus*; Agam: *Anopheles gambiae*). Sequences were aligned and trees were visualized as in Figure 2. Names of sequences include accession numbers.

Our analysis through the databases of arthropod proteins and genes, using as a template both our previously found 14 sequences of *I. scapularis*, as well as that of *C. japonicus*, returned few genes for each species. Figure 6 reports a phylogenetic tree of the NPC2 sequences found in representative insect species and all of those so far found in other arthropods, including the sister groups Tardigrada and Onychophora. We can observe that the number of these proteins in each species of insects is variable, between 2 and 13 in the species where genome information is available. To verify whether these genes were actually expressed at the protein level, we searched through the results of our previous proteome projects (Dani et al., 2011; Iovinella et al., 2011; Mastrobuoni et al., 2013; Zhou et al., 2013), as well as those published by other groups (Chan et al., 2006, 2011, 2013; Sirot et al., 2008; Baer et al., 2009; Swarup et al., 2011). We found that four of the five genes of *A. mellifera* are expressed at the protein level, but only two in the antennae (acc. no. XP_624310; XP_001120140). The products of all these four genes have been detected in several different tissues and organs from individuals of queens, drones and workers, most of them not involved in chemical communication. Moreover, we could not find any of the NPC2 proteins in the antennae nor in other examined tissues of *B. mori*, *A. gambiae*, *L. migratoria*, *A. aegypti*, and *D. melanogaster*, as far as reported in the above cited papers.

In other arthropods, apart from the 14 sequences of *I. scapularis* we found 12 genes encoding NPC2 proteins in *Daphnia pulex* and 11 in the collembolan *Onychiurus arcticus*, besides fewer members in other basal hexapods (Table 1). Orthologs of these proteins also are present in related species, such as *I. ricinus*, *D. magna* and others, but for better clarity we prefer to limit the number of sequences reported in Figure 6 and Table 1 to those of selected species. We also identified 4 sequences in the tardigradan species *Hypsibius dujardini*, 3 in *Limulus polyphemus* (Xiphosura) and one in each of two species of Onychophora, *Peripatopsis sedgwicki* and *Epiparipatus* sp. As these data are based on very limited sequence information for all these species (except for *I. scapularis* and *D. pulex*, whose genome projects have been published), the actual number of NPC2 expressed in each of them could reasonably be higher.

### Three-dimensional structure of NPC2

**Figure 7:**
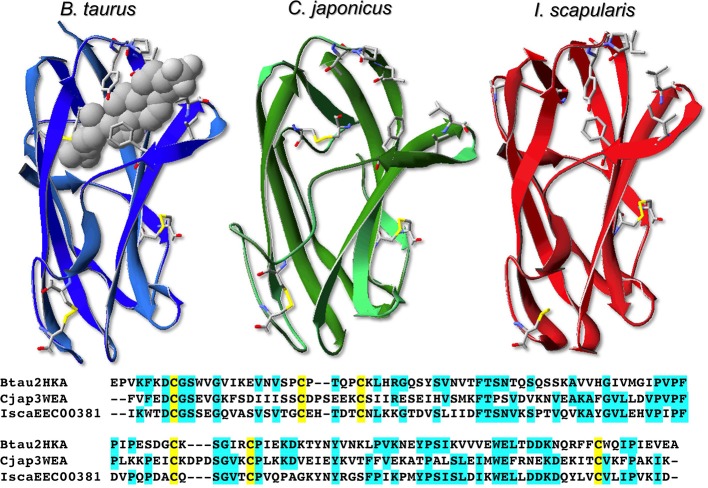
**Three-dimensional structures of bovine (PDB ID: 2HKA) and *Camponotus japonicus* NPC2 (PDB ID: 3WEA), and model of *Ixodes scapularis* NPC2 (acc. no. EEC00381), built on the bovine NPC2 as a template**. The model was obtained using the on-line software “Swiss-Model” (Arnold et al., 2006) and visualized using the Swiss-Model PDB Viewer (Guex and Peitsch, 1997).

The folding of several NPC2 proteins of vertebrate has been solved. It is a very compact and conserved structure resembling a sort of cylindrical basket made of β-sheets and enclosing the binding cavity for cholesterol and other lipids (Figure 7). It reminds in some way of the β-barrel motif of vertebrate OBPs and more in general of lipocalins. Curiously, NPC2 proteins present a conserved motif of six cysteines paired in three disulfide bridges, similarly to insect OBPs, although this might be no more than a coincidence. Figure 7 also reports a model of one of *I. scapularis* proteins, built on the structure of the bovine member (PDB ID: 2HKA), together with the structure of *C. japonicus* NPC2 (PDB ID: 3WEA, Ishida et al., 2014). The entrance to the cavity is gated by a number of amino acids (V59, V64, F66, Y100, P101, I103) conserved or replaced by very similar residues in most of the 14 sequences of *I. scapularis*. These residues are shown in Figure 7. The binding pocket in the model of the tick protein, as in the structure of the bovine one, is lined with a large number of hydrophobic residues.

## Concluding remarks

**Figure 8:**
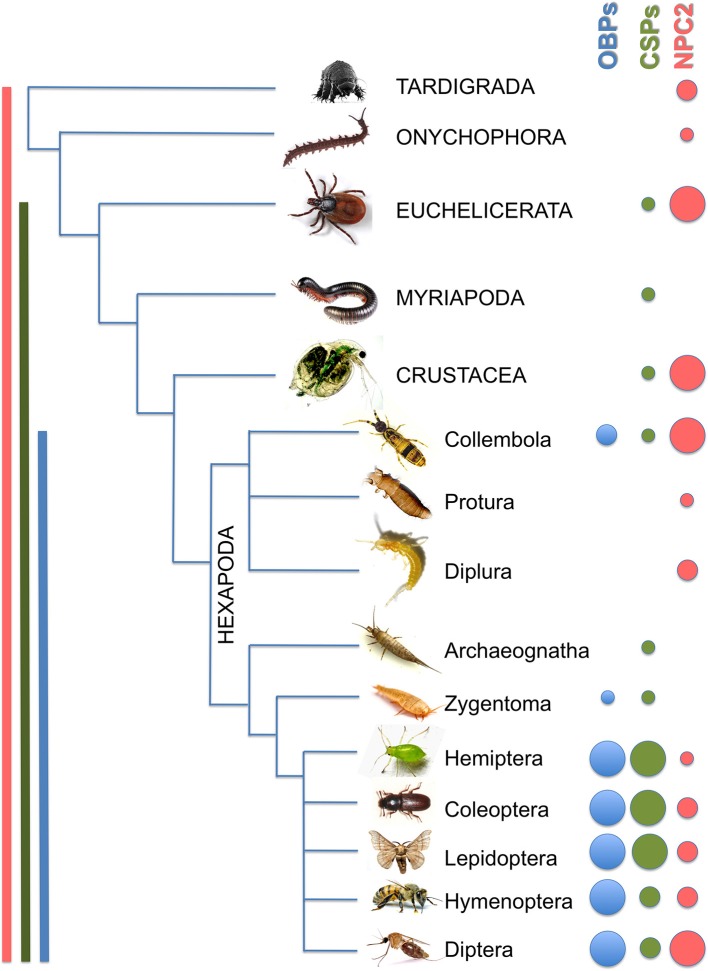
**Overview of OBPs, CSPs and NPC2 genes in arthropods and sister groups**. Taxa are reported in capital letters, Orders in sentence case. Sizes of the dots indicate the maximum number of genes found in each species of the same group (small: 1–2; medium: 3–10; large: >10). Detailed information is reported in Table 1.

In our analysis of soluble proteins likely to be involved in chemical communication across evolution, as summarized in Figure 8, we have found that:

1. OBPs are present in all species of insects so far investigated, including the most primitive ones, but are completely absent in non-insect arthropods.
2. CSPs are more widely expressed and seem to have appeared earlier than OBPs during evolution. In fact, apart from insects, members of this family have been reported in Crustacea, Myriapoda, and Euchelicerata. However, in these groups the small number of genes in each species does not seem to support a function in chemoreception and other roles could be performed by CSPs in non-insect arthropods.
3. We propose that in non-insect arthropods proteins of the NPC2 family might fulfill the role of semiochemical carrier performed by OBPs and CSPs in insects. These proteins are small, soluble and secreted. They present a compact folding resembling under certain aspects the β-barrel of vertebrate OBPs with a binding pocket lined by hydrophobic residues.
4. The relatively large number of NPC2 proteins found in some arthropods and their wide differentiation within the same species suggest that they may have evolved to play a function in binding and discrimination of a variety of semiochemicals.

Certainly the account of soluble proteins of chemoreception in arthropods we have presented in this summary is still fragmentary and incomplete. The fast developing techniques of genome and trascriptome sequencing, as well as proteomic tools, will provide in the near future the necessary information to fill all the gaps and contribute to complete the complex picture of different soluble proteins in chemical communication.

### Conflict of interest statement

The authors declare that the research was conducted in the absence of any commercial or financial relationships that could be construed as a potential conflict of interest.
