# Supplementary material for: Soluble proteins of chemical communication: an overview across arthropods
Source: Front Physiol. 2014 Aug 27;5:320. doi: 10.3389/fphys.2014.00320 (PMC4145409; doi:10.3389/fphys.2014.00320)
Supplement: Supplementary file 1 [file DataSheet1.DOCX]

**Table S1**. Accession numbers of reported sequences. For all the other sequences the names reported in Vieira and Rozas, 2011 have been adopted and accession numbers can be found in their paper.

| **Name** | **Species** | **Order** | **Acc. number** |
| --- | --- | --- | --- |
| FcanOBP1 | *Folsomia candida* | Collembola | GAMN01026304 |
| FcanOBP2 | *Folsomia candida* | Collembola | GAMN01007249 |
| FcanOBP3 | *Folsomia candida* | Collembola | GAMN01021378 |
| FcanOBP4 | *Folsomia candida* | Collembola | GAMN01004422 |
| FcanOBP5 | *Folsomia candida* | Collembola | GAMN01001625 |
| OcinOBP1 | *Orchesella cincta* | Collembola | GAMM01008770 |
| OcinOBP2 | *Orchesella cincta* | Collembola | GAMM01000249 |
| TaurOBP1 | *Tricholepisma aurea* | Zygentoma | CV177751 |
| CantCSP1 | *Cryptopygus antarcticus* | Collembola | FF278675 |
| CantCSP2 | *Cryptopygus antarcticus* | Collembola | FF278733 |
| AmarCSP1 | *Anurida maritima* | Collembola | FN193420 |
| FcanCSP1 | *Folsomia candida* | Collembola | EV477937 |
| TaurCSP1 | *Tricholepisma aurea* | Zygentoma | CV177502 |
| LysiCSP1 | *Lepismachilis y-signata* | Archaeognatha | FN224208 |
| LysiCSP2 | *Lepismachilis y-signata* | Archaeognatha | FN221262 |
| DpulCSP1 | *Daphnia pulex* | Branchiopoda | DQ855479 |
| TcanCSP1 | *Triops cancriformis* | Branchiopoda | FM870352 |
| TcanCSP2 | *Triops cancriformis* | Branchiopoda | FM868683 |
| AfraCSP1 | *Artemia franciscana* | Branchiopoda | ES494687 |
| IscaCSP1 | *Ixodes scapularis* | Acari | DQ855478 |
| JulCSP1 | *Julida sp.* | Diplopoda | CV178590 |
| JulCSP2 | *Julida sp.* | Diplopoda | CV178605 |
| AgigCSP1 | *Archispirostreptus gigas* | Diplopoda | FN195485 |
| AgigCSP2 | *Archispirostreptus gigas* | Diplopoda | FN195686 |
